# Orlistat and ezetimibe could differently alleviate the high-fat diet-induced obesity phenotype by modulating the gut microbiota

**DOI:** 10.3389/fmicb.2022.908327

**Published:** 2022-08-15

**Authors:** Jin Jin, Jiani Wang, Ruyue Cheng, Yan Ren, Zhonghua Miao, Yating Luo, Qingqing Zhou, Yigui Xue, Xi Shen, Fang He, Haoming Tian

**Affiliations:** ^1^Department of Endocrinology and Metabolism, West China Hospital of Sichuan University, Chengdu, Sichuan, China; ^2^Department of Nutrition and Food Hygiene, West China School of Public Health and West China Fourth Hospital, Sichuan University, Chengdu, Sichuan, China; ^3^Frontier Medical Service Training Battalion of Army Military Medical University, Changji Hui Autonomous Prefecture, Xinjiang, China

**Keywords:** gut microbiota, SCFAs, orlistat, ezetimibe, obesity

## Abstract

This study aimed to evaluate the possible anti-obesity effects of orlistat and ezetimibe and determine the mechanism by which they alter the composition of gut microbiota and short-chain fatty acids (SCFAs) in mice with a high-fat diet (HFD)-induced obesity. Eighty male, specific pathogen-free C57BL/6J mice aged 3 weeks were divided into four groups (*n* = 20). The NCD group was fed with a normal diet, and the HFD, HFD+ORL, and HFD+EZE groups were fed with HFD for 20 weeks. From the 13th week onward, the HFD+ORL and HFD+EZE groups were administered with orlistat and ezetimibe, respectively. The glucose and lipid metabolism of the tested mice were evaluated by analyzing blood biochemical indicators during the intervention. Furthermore, the changes in the structure of the fecal microbiota and the fecal SCFA content were analyzed by 16S rRNA sequencing and gas chromatography-mass spectrometry, respectively. HFD induced the obesity phenotype in mice. Compared to the HFD group, the body weight, visceral fat-to-body weight ratio, serum total cholesterol (TC), high-density lipoprotein-cholesterol (HDL-C), and oral glucose tolerance test (OGTT) of the HFD+ORL group significantly decreased, whereas fecal butyric acid levels significantly increased. Ezetimibe intervention significantly reduced the OGTT, serum TC, and HDL-C levels only. The α-diversity of the gut microbiota significantly decreased after intervention with orlistat and ezetimibe. Orlistat altered the relative abundance of some bacteria in the fecal microbiota. The populations of *Firmicutes, Alistipes*, and *Desulfovibrio* decreased, whereas those of *Verrucomicrobia* and *Akkermansia* significantly increased. Ezetimibe caused changes only in some low-abundance bacteria, as manifested by a decrease in *Proteobacteria* and *Desulfovibrio*, and an increase in *Bacteroides*. The administration of orlistat and ezetimibe can characteristically influence the body weight and serum lipid metabolism, and glucolipid levels in diet-induced obese mice and is accompanied by significant changes in the gut microbiota and SCFAs. These results suggest that the two drugs might exert their own specific anti-obesity effects by modulating the gut microbiota in a different manner. The enhanced health-promoting effect of orlistat might result from its stronger ability to alter the gut microbiota and SCFAs, at least partly.

## Graphical Abstract

OGTT, oral glucose tolerance test; TC, total cholesterol; LDL-C, low-density lipoprotein-cholesterol; HDL-C, high-density lipoprotein-cholesterol. 
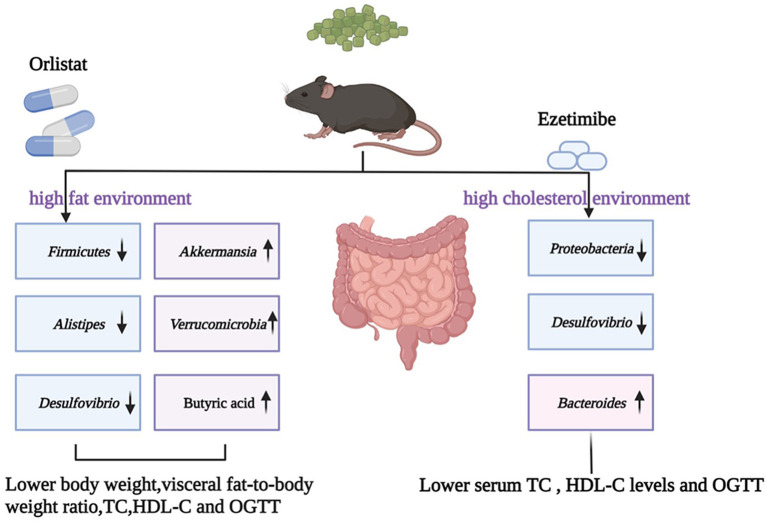

## Introduction

Over the past few decades, obesity has become a growing global public health problem. Nearly 2 billion adults worldwide are overweight, and more than half of them are classified as obese (Hoffman et al., 2021). According to the World Health Organization statistics, at least 280,000 people died due to overweight or obesity (Hussain et al., 2020). The Report on Nutrition and Chronic Disease Status of Chinese Residents (2020) showed that the overweight and obesity rate of Chinese adult residents exceeded 50%. Based on current trends, the projections suggested an increase in the prevalence of obesity to 40% in adult women, 60% in adult men, and 25% in children by the year 2050 (Milano et al., 2020). Obesity is often accompanied by other metabolic diseases, such as hyperlipidemia, hypercholesterolemia, cardiovascular disease, liver steatosis, and type 2 diabetes mellitus (T2DM), all of which increase the risk of death (Mayoral et al., 2020). Therefore, the effective control of overweight and obesity is of great significance to preventing and controlling chronic noncommunicable diseases in the world, particularly in China.

Although the influence of diet, lifestyle, and genetics on the occurrence and progression of obesity is well-known, new research suggests that the gut microbiota may be involved in the pathogenesis of obesity. Observations made in the past 20 years indicated that the gut microbiota might contribute to the metabolic health of the human host. When the intestinal microbiota is abnormal, it may cause various common metabolic disorders, including obesity, T2DM, nonalcoholic liver disease, and dyslipidemia (Fan and Pedersen, 2021). The majority of microorganisms that inhabit humans reside within the intestines and are influenced by the host's mode of birth, lifestyle, medication, and genetics (Lynch and Pedersen, 2016). As early as 2005, Gordon's research team discovered that obesity would change the ecology of the gut microbiota, and the obvious gut microbial feature of obese people shows a significant increase in *Firmicutes*/Bacteroides (Ley et al., 2005; Voigt et al., 2014). Since then, more and more evidence has shown that interventions aimed at regulating the intestinal microbiota (such as probiotics, prebiotics, and fecal microbiota transplantation) are effective and have a comprehensive effect on obesity (Lynch and Pedersen, 2016; López-Moreno et al., 2020; Sergeev et al., 2020; Yu et al., 2020).

Oral medication is one of the approaches to fight obesity and its complications in humans. In clinical practice, orlistat or ezetimibe is often used to treat obesity or hypercholesterolemia. Orlistat is an oral over-the-counter anti-obesity drug approved by the US Food and Drug Administration for chronic weight management. Studies have shown that orlistat loses 2.9% of the total body weight after subtracting a placebo for at least 12 months (Tak and Lee, 2021). Orlistat induces weight loss by inhibiting lipase in the mucosa of the stomach, small intestine, and pancreas, thereby preventing triglycerides (TGs) from being broken down into fatty acids and absorbed in the intestine (Son and Kim, 2020). Ezetimibe is a lipid-lowering drug that inhibits the intestinal absorption of dietary and bile cholesterol without affecting the absorption of fat-soluble nutrients. It is clinically used to treat hypercholesterolemia (Kosoglou et al., 2005). Both drugs act on the gastrointestinal tract, causing TGs or cholesterol to accumulate in the intestinal tract, respectively (Lynch and Pedersen, 2016). A growing number of studies have shown that the gut microbiota is involved in the metabolism of many drugs and can modulate their effectiveness and side effects (Zimmermann et al., 2019; Klünemann et al., 2021).

The main nutrient source of intestinal microbes is complex carbohydrates that are not digested by upstream digestive tract enzymes. These carbohydrates can be fermented by the intestinal microbiota to produce metabolites, such as short-chain fatty acids (SCFAs). Previous studies have shown that a high-fat diet (HFD) and a high-cholesterol diet can change the gut microbiota differently and its metabolites, including SCFAs and bile acids (Liang et al., 2021). Moreover, orlistat and ezetimibe have limited effects on the gut microbiota of obese patients in Xinjiang in a previous study (Jin et al., 2021). At present, little is known about the identity of the bacterial population that ultimately participates in lipid and cholesterol metabolism, and its underlying mechanism is still unclear.

Therefore, this study selected an HFD to induce obesity in mice and used orlistat and ezetimibe to intervene in obese mice. The changes in body weight, blood glucose, blood lipids, liver function, intestinal microbiota, and other indicators were observed to find the core microorganisms that respond to orlistat and ezetimibe and determine the contribution of the gut microbiota to host metabolism.

## Materials and methods

### Mice

Eighty male, specific pathogen-free C57BL/6J mice aged 3 weeks were purchased from Beijing Huafukang Bioscience Co., Ltd. (approval no. SCXK2019-0008) and kept at the Laboratory Animal Center of West China Second Hospital of Sichuan University (approval no. SYXK2018-209) at an ambient temperature of 20–26°C, relative area minimum static pressure difference of 10 Pa, noise ≤ 60 dB (a), day and night alternate time of 12 h, and free drinking water and feed. This experimental design was approved by the Ethics Committee of West China Second Hospital of Sichuan University. All experimental procedures were carried out in accordance with the guidelines for animal experiments of West China School of Public Health of Sichuan University and the guidelines of the Experimental Animal Center of West China Second Medical College of Sichuan University.

### HFD feed and orlistat and ezetimibe treatment

Eighty 3-week-old male C57BL/6J mice were randomly divided into four groups with 20 mice in each group: NCD, HFD, HFD+ORL, and HFD+EZE groups. The NCD group was fed a normal diet, and the other three groups were fed with HFD (D12492; Research Diets, New Brunswick, NJ, USA). From the 13th week onward, the HFD+ORL group was given orlistat (approval no. H20123131; Zhien Pharmaceutical, Chongqing, China) by gavage, and the HFD+EZE group was given ezetimibe (approval no. H20160181; MSD Pharma Pte. Ltd., Gateway West Singapore) by gavage until the 20th week. The dose of ezetimibe and orlistat was 10 and 120 mg/kg/day, respectively. The drug was dissolved in normal saline and intragastrically administered at 0.2 ml each time, once daily. The NCD and HFD groups were given the same volume of normal saline.

### Body weight, organ indexes, and visceral fat-to-body weight ratio

The body weight of mice was measured once weekly until the end of the experiment. Mice were anesthetized by CO_2_ and killed by cervical dislocation. The liver, spleen, pancreas, gonadal fat, perirenal fat, and mesenteric fat were collected and weighed. The organ indexes and visceral fat-to-body weight ratio of mice were calculated. Organ index=organ weight (mg)/body weight of mice (g). Visceral fat-to-body weight ratio=fat weight (g)/mice body weight (g) ×100%.

### Fasting blood glucose (FBG) and oral glucose tolerance test (OGTT)

Fasting blood glucose was measured once monthly. After fasting for 12 h, FBG was measured by using Roche Accu-Chek Active test strips (approval no. 20182401933; Roche Diabetes Care GmbH, Mannheim, Germany). OGTT was determined before and at the end of the experiment. After fasting for 12 h, FBG (0 min) was determined by a Roche blood glucose meter, and each mouse was given a 2.0 g/kg glucose solution quickly. The blood glucose values at 30, 60, 90, and 120 min after administration were determined, and the area under the blood glucose curve (AUC) was calculated. AUC=15×(GLU0 + 2GLU30 + 2GLU60 + 2GLU90+GLU120).

### Detection of serum biochemical indicators, insulin, leptin, and adiponectin

At the end of the experiment, blood samples were collected and kept at room temperature for 2 h. Blood was centrifuged at 2,000 × *g* for 20 min, and the supernatant was absorbed and centrifuged again for 5 min to obtain the serum. The serum was kept at −80°C for later use. The serum total cholesterol (TC), TG, high-density lipoprotein-cholesterol (HDL-C), low-density lipoprotein-cholesterol (LDL-C), alanine aminotransferase (ALT), aspartate transaminase (AST), total bilirubin (TBIL), direct bilirubin (DBIL), albumin (ALB), and γ-glutamyl transpeptidase (γ-GT) were detected by Wuhan Servicebio Biotechnology Co., Ltd. Mouse Ins1/insulin-1 enzyme-linked immunosorbent assay (ELISA) kit (Millipore), mouse/rat leptin Quantikine ELISA kit (R&D Systems), and mouse adiponectin/Acrp30 Quantikine ELISA kit (R&D Systems) were used to determine the serum insulin, leptin, and adiponectin content in strict accordance with the instructions provided in the kit.

### DNA extraction of fecal bacteria

At the end of the experiment, a sterile stool collection box was used to collect the fecal samples of mice. The samples were stored in a sterile EP tube and preserved at −80°C. Fecal bacterial DNA was extracted according to the TIANamp fecal DNA kit (Tiangen Biotech Co., Ltd., Beijing, China).

### 16S rRNA encoding gene sequencing and bioinformatics analysis

To ensure the accuracy of high-throughput sequencing results, the extracted fecal DNA was tested for purity and concentration and sent to Chengdu Basebio Biotechnology Co., Ltd. for high-throughput sequencing with Illumina MiSeq (Illumina, Inc., Foster City, CA, USA). Illumina high-throughput sequencing results were converted into raw sequencing data after base calling using bcl2fastq (version 1.8.4). Reads with missing barcodes, incorrect barcodes, or conflicting barcode pairs were discarded, and mismatches of more than three per primer were cut off using Trimmomatic (version 0.36). Splice double-ended sequences were analyzed using FLASH (version 1.2.11), which should overlap by at least 10 base pairs, and a maximum of two base mismatches was allowed. After this, the sequences were defined as high-quality sequences.

Chimeric sequences were detected and removed, and the operational taxonomic unit (OTU) was clustered using Usearch (version 11) UNOISE3 algorithm. Usearch was used to compare to the RDP (Release_16) database, and OTU sequences were annotated to obtain taxonomy information for each OTU. Taxonomic assignments were considered reliable when bootstrap confidence values exceeded 0.75. Phylogenetic tree construction was conducted using the USEARCH cluster_aggd command.

Subsequently, the α-diversity indexes (Observed_OTUs, Chao1, ACE, Fisher, Shannon, and Simpson indexes) were calculated based on the species abundance of each sample in the OTU list using the summary.single command in Mothur. The β-diversity was identified by a principal coordinates analysis (PCoA) using R language PCoA for statistical analysis and graphing. Colony structure statistical analysis and species richness analysis used QIIME to generate species abundance tables and multisample species distribution maps at different taxonomic levels (phylum, class, order, family, and genus) based on the results of the OTU table. Finally, R (version 3.4.1) was used to visualize the statistical results.

### Fecal SCFA analysis

Fecal SCFA analysis was conducted using gas chromatography-mass spectrometry (GC-MS). An Agilent HP-INNOWax GC-MS (Agilent Technologies, Inc., Santa Clara, CA, USA) was used for quantification. To 50 mg of sample, 50 μL of 15% phosphoric acid (China National Pharmaceutical Group Co., Ltd., Beijing, China), 100 μL of 125 μg/mL internal standard solution (isohexanoic acid, >98%; Sigma), and 400 μL of ether (China National Pharmaceutical Group) were added and homogenized for 1 min and centrifuged at 4°C at 12,000 rpm for 10 min, and the supernatant was taken for the test. Acetic acid (AA, >99.5%), propionic acid (PA, >99.0%), isobutyric acid (IBA, >99.0%), butyric acid (BA, >99.0%), isovaleric acid (IVA, >99.0%), and valeric acid (VA, >98.0%) were purchased from Sigma. Caproic acid (CA, ≥99.5%) was purchased from Aladdin Shanghai Biochemical Technology (Shanghai, China).

### Statistical analysis

The IBM SPSS Statistics software package version 19.0 (Statistical Package for the Social Sciences, Chicago, IL, USA) and GraphPad Prism 8.0 (GraphPad Software, Inc., San Diego, CA, USA) were used to analyze the experimental data. The experimental results were expressed as the mean ± standard deviation. One-way analysis of variance or the Kruskal–Wallis nonparametric test was used to compare multiple groups of independent samples, followed by a *post-hoc* least significant difference and Bonferroni test. All tests were two-tailed. *P* < 0.05 indicated a statistically significant difference. The correlations between the abundance of key OTUs and phenotypes were assessed using Spearman's correlation analysis.

## Results

### Body weight, organ index, and visceral fat-to-body weight ratio

**Figure 1:**
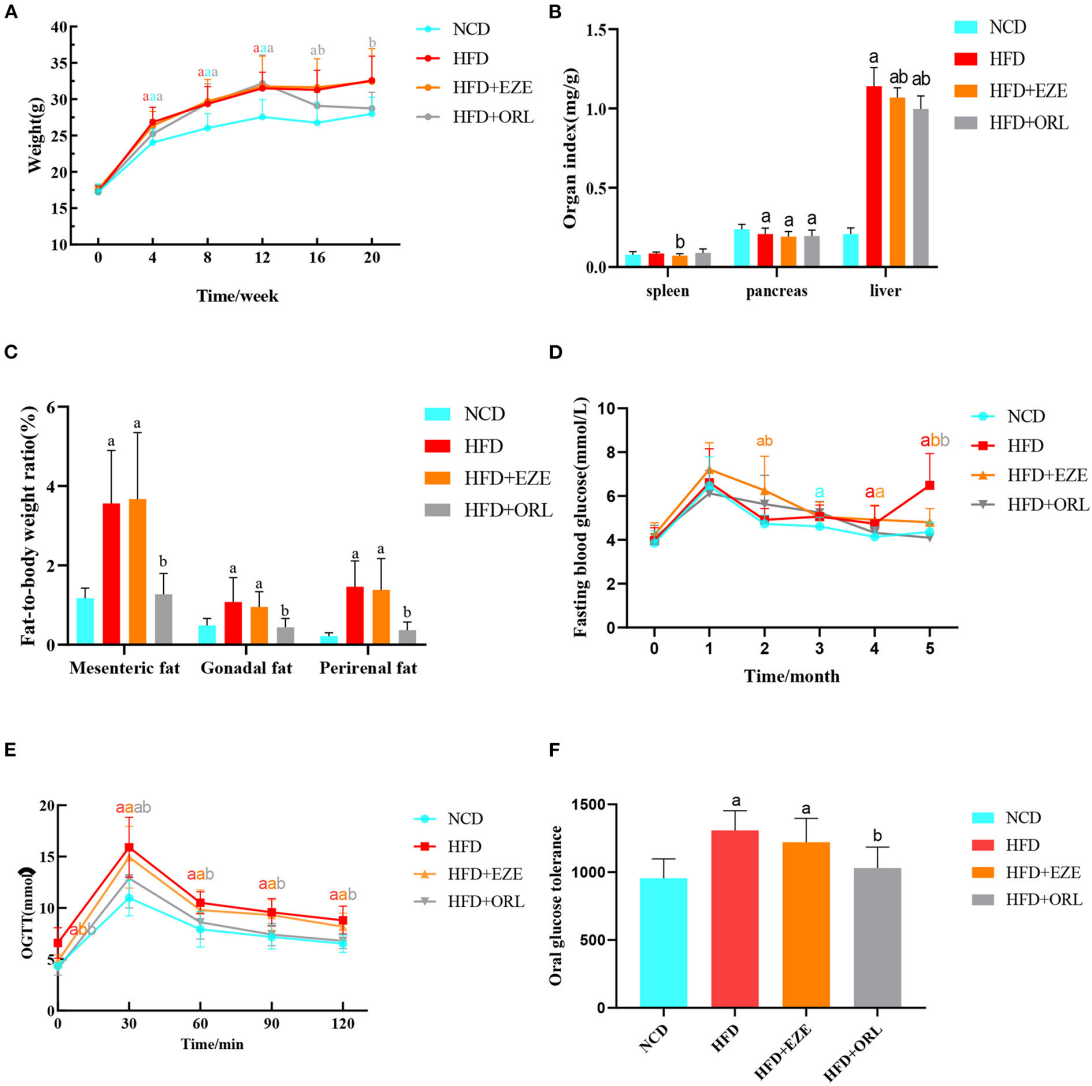
Body weight, organ indexes, visceral fat-to-body weight ratio, and the glucose response of mice. **(A)** The body weight of mice in different groups. **(B)** Organ (spleen, pancreas, and liver) indexes of mice. **(C)** Visceral fat-to-body weight ratios of mice. **(D)** Determination of fasting blood glucose levels of mice each month. **(E)** An oral glucose tolerance test (OGTT) was performed on mice in the 20th week. **(F)** Area under the oral glucose tolerance curve of mice. a: *P* < 0.05 compared with the NCD group. b: *P* < 0.05 compared with the HFD group. a in red: HFD group compared with NCD group. a in orange: HFD+EZE group compared with NCD group. a in gray: HFD+ORL group compared with NCD group. b in orange: HFD+EZE group compared with HFD group. b in gray: HFD+ORL group compared with HFD group. NCD, normal diet; HFD, high-fat diet; HFD+EZE, high-fat diet and intervention of ezetimibe; HFD+ORL, high-fat diet and intervention of orlistat; *n* = 19-20 per group.

In the first 12 weeks of the experiment, the body weight of the HFD, HFD+EZE, and HFD+ORL groups was significantly higher than the NCD group. After orlistat intervention, the body weight of the HFD+ORL group was significantly lower than the HFD group. However, there was no significant difference in body weight between the HFD and HFD+EZE groups after ezetimibe intervention (Figure 1A). The spleen index of the HFD+EZE group was lower than the NCD group. The pancreas index of the HFD, HFD+EZE, and HFD+ORL groups was lower than the NCD group. The liver index of the HFD, HFD+EZE, and HFD+ORL groups was significantly lower than the NCD group (Figure 1B). Visceral fat tissue accumulation (including the mesenteric fat tissue, gonadal fat tissue, and the perirenal fat tissue) in the HFD and HFD+EZE groups was greater than in the NCD group. However, visceral fat accumulation in the HFD+ORL group was significantly less than in the HFD group (Figure 1C).

### Alterations in blood glucose levels

There was no significant difference in FBG levels between groups at the 0th and 1st month of the experiment. On the 3rd, 4th, and 5th months of the experiment, the FBG levels of the HFD group were higher than the NCD group. A month after ezetimibe intervention (4th month), the FBG levels of the HFD+EZE group were still significantly higher than the NCD group, which decreased significantly in the 5th month and were lower than the HFD group and similar to the NCD group. Two months after orlistat intervention (5th month), the FBG levels of the HFD+ORL group were significantly lower than the HFD group, which was similar to the NCD group (Figure 1D).

As shown in Figure 1E, the blood glucose of the four groups increased to the highest levels at 30 min, and the blood glucose levels of the HFD and HFD+EZE groups were significantly higher than the NCD group at all time points. However, the blood glucose level of the HFD+ORL group was lower than the HFD group at all time points. Moreover, the AUC of the HFD and HFD+EZE groups was significantly higher than the NCD group, and the AUC of the HFD+ORL group was significantly lower than the HFD group and similar to the NCD group (Figure 1F).

### Detection of serum lipid and hormones

**Figure 2:**
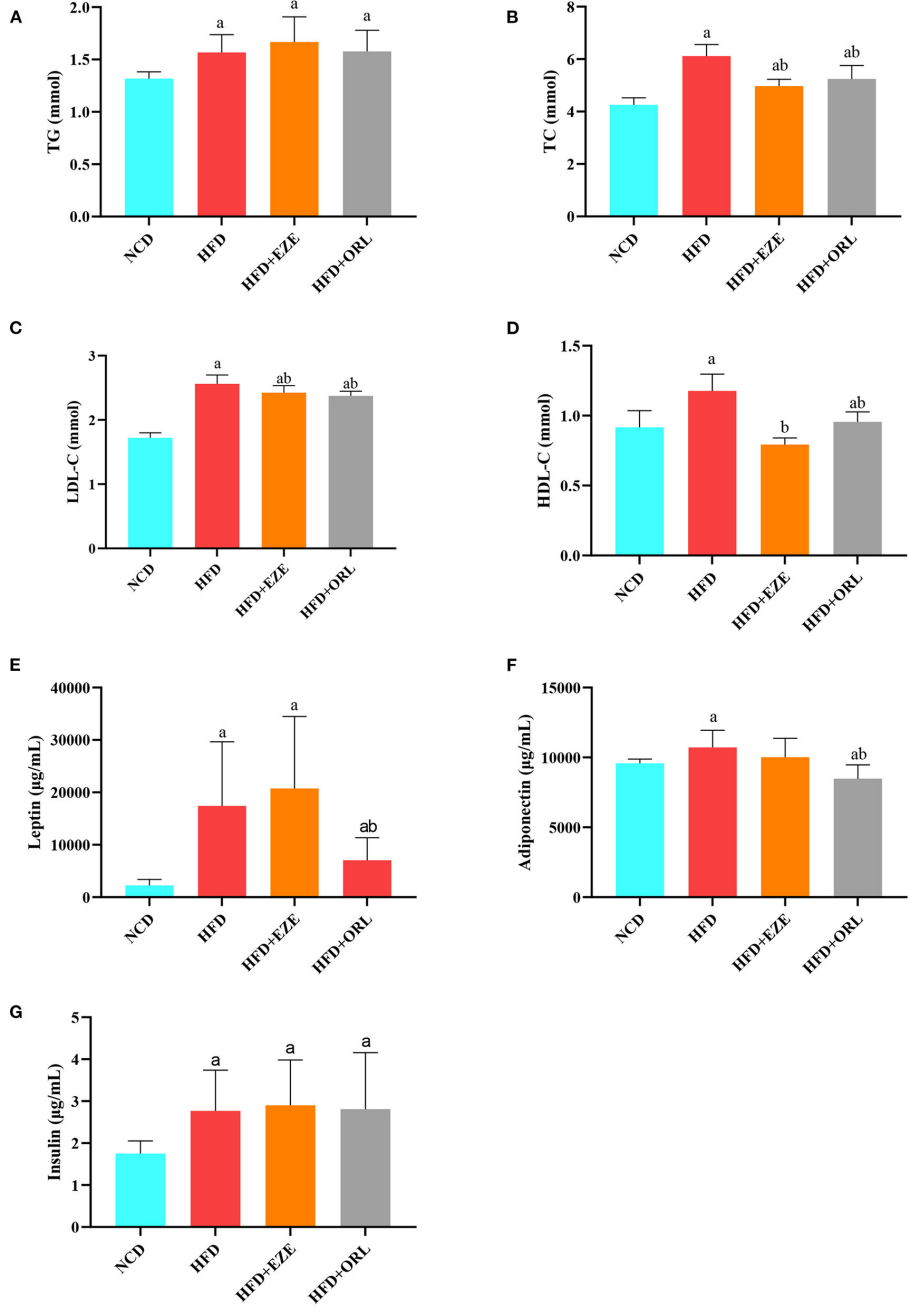
Serum biochemical parameters of mice at week 20. **(A)** Triglyceride (TG) levels. **(B)** Total cholesterol (TC) levels. **(C)** Low-density lipoprotein-cholesterol (LDL-C) levels. **(D)** High-density lipoprotein-cholesterol (HDL-C) levels. **(E)** Leptin levels. **(F)** Adiponectin levels. **(G)** Insulin levels. a: *P* < 0.05 compared with the NCD group. b: *P* < 0.05 compared with the HFD group. NCD, normal diet; HFD, high-fat diet; HFD+EZE, high-fat diet and intervention of ezetimibe; HFD+ORL, high-fat diet and intervention of orlistat; *n* = 10 per group.

The blood lipid composition of each group is shown in Figure 2. After 20 weeks of intervention, the serum TG, TC, LDL-C, and HDL-C levels of HFD-fed mice were significantly higher than mice fed with a normal diet (Figures 2A–D). After orlistat and ezetimibe intervention, compared to the HFD group, no significant difference was found in TG and LDL-C; however, the serum TC and HDL-C levels significantly decreased (Figures 2A–D). As shown in Figures 2E–G, the serum leptin, adiponectin, and insulin levels in HFD-fed mice were significantly higher than in the NCD group. There was no significant difference in the serum leptin, adiponectin, and insulin levels between the HFD and HFD+EZE groups. Conversely, the serum leptin and adiponectin levels in the HFD+ORL group were significantly lower than in the HFD group, although the serum insulin levels between the HFD and HFD+ORL groups were similar.

### Detection of liver function

**Figure 3:**
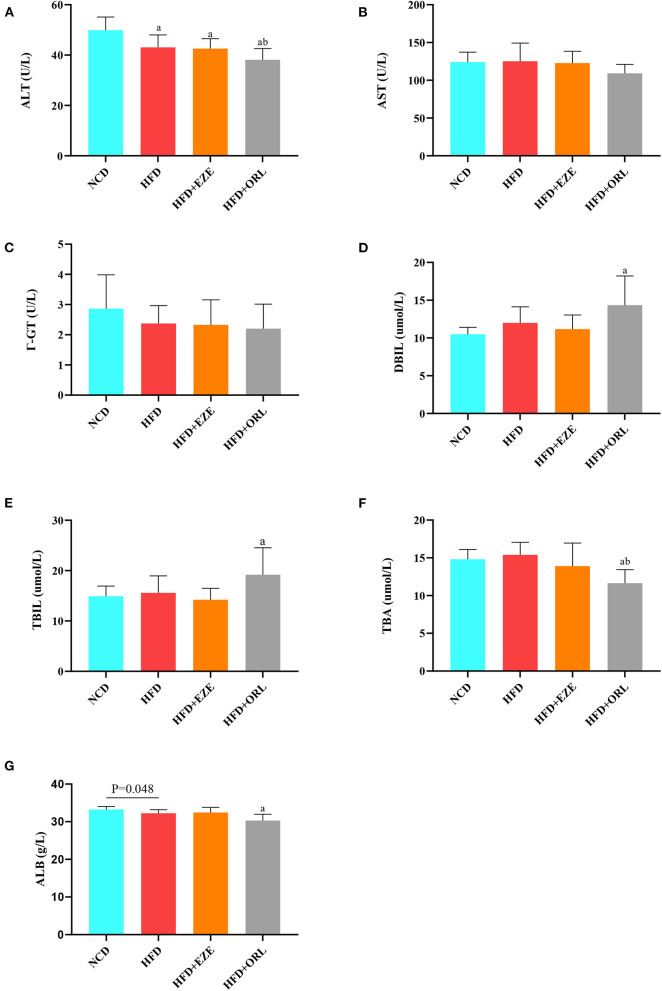
Liver function levels of mice in different groups. **(A)** Serum alanine aminotransferase (ALT) levels; **(B)** serum aspartate aminotransferase (AST) levels; **(C)** serum Γ-glutamyl transpeptidase (Γ-GT) levels; **(D)** serum direct bilirubin (DBIL) levels; **(E)** serum total bilirubin (TBIL) levels; **(F)** serum total bile acid (TBA) levels; and **(G)** serum albumin (ALB) levels. a: *P* < 0.05 compared with the NCD group. b: *P* < 0.05 compared with the HFD group. NCD, normal diet; HFD, high-fat diet; HFD+EZE, high-fat diet and intervention of ezetimibe; HFD+ORL, high-fat diet and intervention of orlistat; *n* = 10 per group.

The serum liver function indexes of mice are shown in Figure 3. The serum ALT levels in HFD-fed mice were significantly lower than in mice fed with a normal diet, and the ALT levels in the HFD+ORL group were significantly lower than in the HFD group (Figure 3A). However, there were no significant differences in AST and γ-GT among groups (Figures 3B,C). Besides, the serum DBIL and TBIL levels in the HFD+ORL group were significantly higher than in the NCD group (Figures 3D,E). Moreover, the serum TBA levels in the HFD+ORL group were significantly lower than in the NCD and HFD groups (Figure 3F). The serum ALB levels were significantly lower than in the NCD group (Figure 3G).

### Fecal SCFA analysis

**Figure 4:**
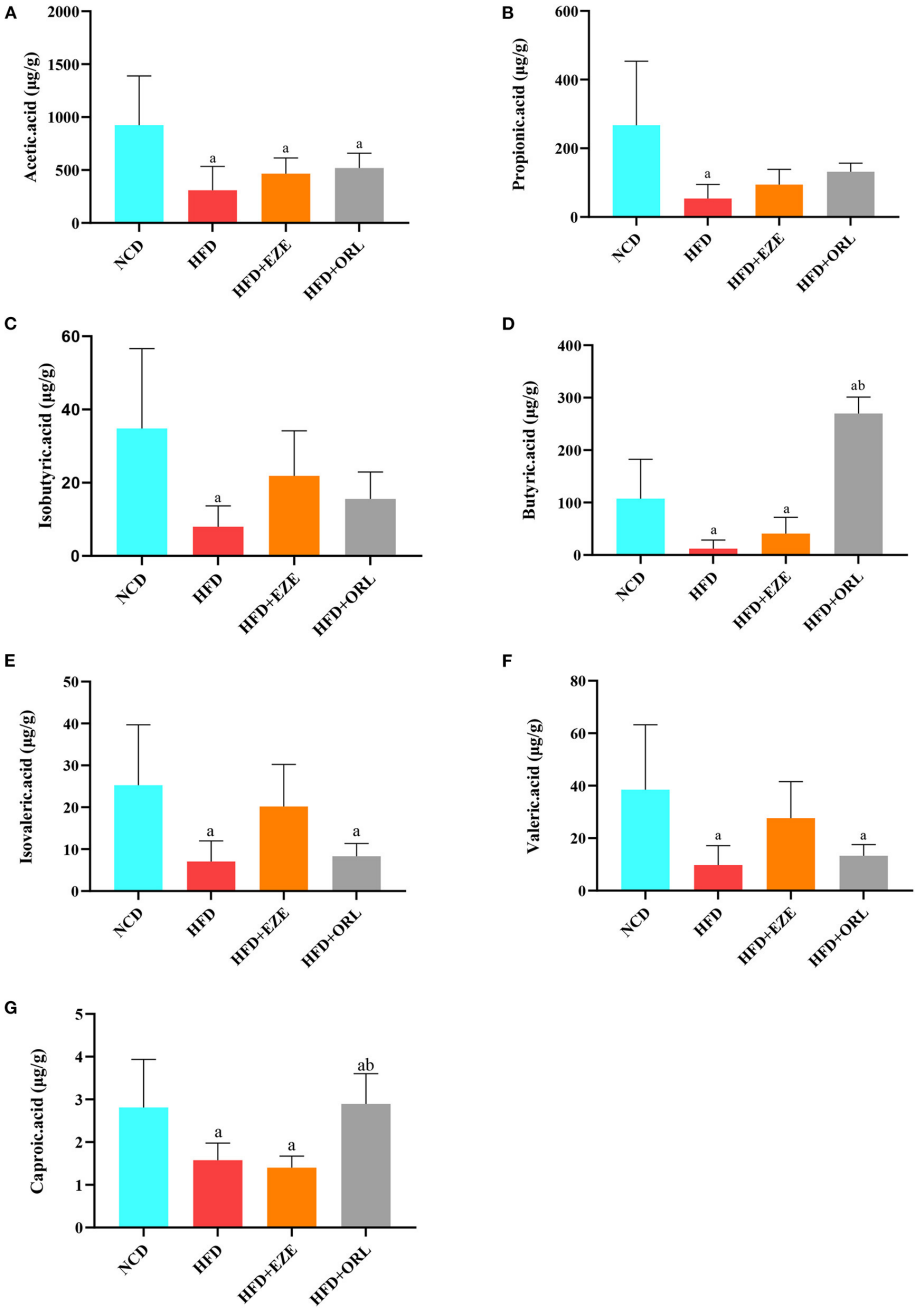
Fecal short-chain fat acid (SCFA) levels of mice in different groups (μg/g wet weight of feces). **(A)** Acetic acid levels in fecal samples. **(B)** Propionic acid levels in fecal samples. **(C)** Fecal isobutyric acid levels. **(D)** Fecal butyric acid levels. **(E)** Fecal isovaleric acid levels. **(F)** Fecal valeric acid levels. **(G)** Fecal caproic acid levels. a: *P* < 0.05 compared with the NCD group. b: *P* < 0.05 compared with the HFD group. NCD, normal diet; HFD, high-fat diet; HFD+EZE: high-fat diet and intervention of ezetimibe; HFD+ORL: high-fat diet and intervention of orlistat; *n* = 6 per group.

Compared to the NCD group, AA, PA, BA, IBA, and VA in the HFD group significantly decreased, AA in the HFD+EZE and HFD+ORL groups significantly decreased, BA in the HFD+EZE group significantly decreased, and VA and IVA in the HFD+ORL group significantly decreased. Compared to the HFD group, SCFAs in the HFD+EZE group did not change significantly (Figures 4A–G). However, BA and CA in the HFD+ORL group significantly increased, which was significantly higher than in the HFD groups (Figures 4D,G).

### Modifications in the composition of the fecal microbiota after orlistat treatment

**Figure 5:**
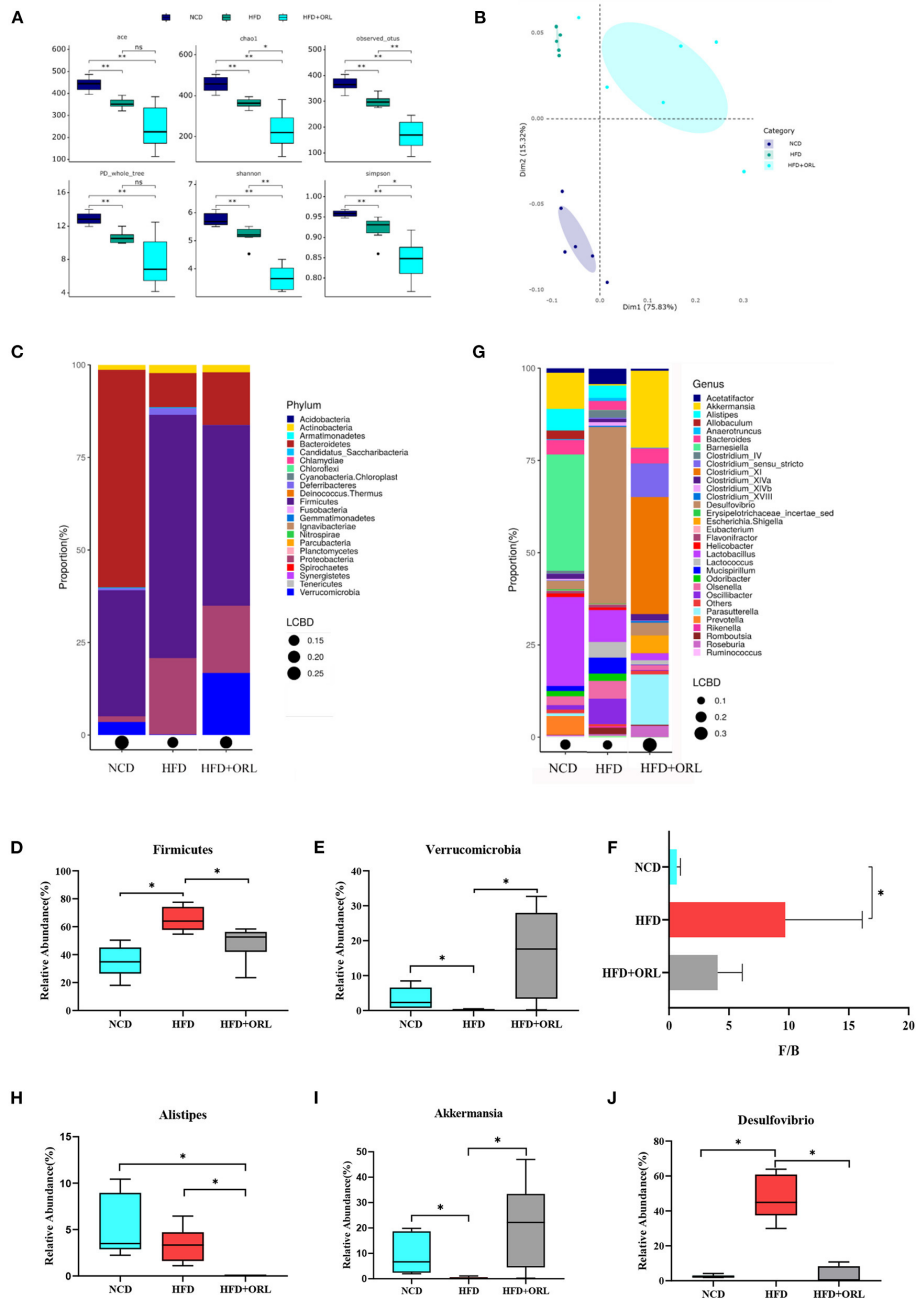
Alterations in gut microbiota before and after orlistat intervention. **(A)** The α-diversity of fecal microbiota in mice. **(B)** Beta-diversity was visualized by PCoA analysis. **(C)** Bar plot of gut microbiota composition at the phylum level. **(D,E)** The relative abundance of *Firmicutes* and *Verrucomicrobia* was significantly changed after intervention by Metastas analysis. **(F)** *Firmicutes*-to-*Bacteroidetes* ratio (*F*/*B*) of mice. (G) Bar plot of gut microbiota composition at the genus level. **(H–J)** The relative abundance of *Alistipes, Akkermansia*, and *Desulfovibrio* was significantly changed after orlistat intervention by Metastas analysis. *p < 0.05. LCBD, Local contributions to beta-diversity; LCBD values represent the degree of uniqueness of the sampling units in terms of community composition. NCD, normal diet; HFD, high-fat diet; HFD+ORL, high-fat diet and intervention of orlistat; *n* = 6 per group.

The α-diversity indexes of HFD-fed mice, including ACE, Chao1, Observed_OTUs, Shannon, Simpson, and PD_whole_tree index, were all significantly decreased compared to mice fed with a normal diet. After orlistat intervention, the Chao1, Observed_OTUs, Shannon, and Simpson indexes were significantly lower than the HFD group (Figure 5A). The PCoA analysis based on the weighted Unifrac distance showed that the principal component 1 separated the NCD group from the HFD group and the HFD+ORL component, and its interpretation was 75.82%. The HFD and HFD+ORL groups were not completely separated, and the axis contribution rate was 15.32% (Figure 5B). The PERMANOVA analysis also showed that the beta-diversity of the HFD+ORL group was significantly different from the remaining two groups (Table 1).

**Table 1:** PERMANOVA analysis of gut microbiota based on weighted Unifrac distance.

**Groups compared**	***P*-values**	***P*-adjust**
HFD+ORL vs. HFD	0.00	0.01*
HFD+ORL vs. NCD	0.01	0.01*
HFD vs. NCD	0.01	0.01*

* P < 0.05. NCD, normal diet; HFD, high-fat diet; HFD+ORL, high-fat diet and intervention of orlistat; subject to the adjusted P-value, n = 6 per group.

After 8 weeks of orlistat intervention, at the phylum level, the five main bacterial phyla in the NCD group were *Bacteroidetes, Firmicutes, Verrucomicrobia, Proteobacteria*, and *Actinobacteria* in order of relative abundance; the five main bacterial phyla of the HFD group were *Firmicutes, Proteobacteria, Bacteroidetes, Actinobacteria*, and *Deferribacteres*; and the five main bacterial phyla of the HFD+ORL group were *Firmicutes, Proteobacteria, Verrucomicrobia, Bacteroidetes, and Actinobacteria* (Figure 5C). Compared to the NCD group, *Firmicutes* in the HFD group significantly increased, and *Verrucomicrobia* significantly decreased; however, compared to the HFD group, *Firmicutes* in the HFD+ORL group significantly reduced (Figure 5D), and *Verrucomicrobia* significantly increased (Figure 5E). After HFD intervention, the *F*/*B* of the HFD group increased significantly. After orlistat intervention, although the *F*/*B* value showed a decreasing trend, the difference was not statistically significant (Figure 5F).

At the genus level, the five major bacterial genera in the NCD group were *Barnesiella, Lactobacillus, Akkermansia, Alistipes*, and *Prevotella* in order of relative abundance; the five major bacterial genera in the HFD group were *Desulfovibrio, Lactobacillus, Oscillibacter, Olsenella*, and *Lactococcus*; and the five main bacterial genera in the HFD+ORL group were *Clostridium XI, Akkermansia, Parasutterella, Clostridium sensu stricto*, and *Escherichia*/*Shigella* in order (Figure 5G). Compared to the NCD group, the relative abundance of *Alistipes* and *Akkermansia* significantly decreased (Figures 5H,I), and the relative abundance of *Desulfovibrio* significantly increased in the HFD group (Figure 5J); however, compared to the HFD group, the relative abundance of *Alistipes* and *Desulfovibrio* in the HFD+ORL group significantly decreased (Figures 5H,J), and the relative abundance of *Akkermansia* significantly increased (Figure 5I).

### Modifications in the composition of the fecal microbiota after ezetimibe treatment

**Figure 6:**
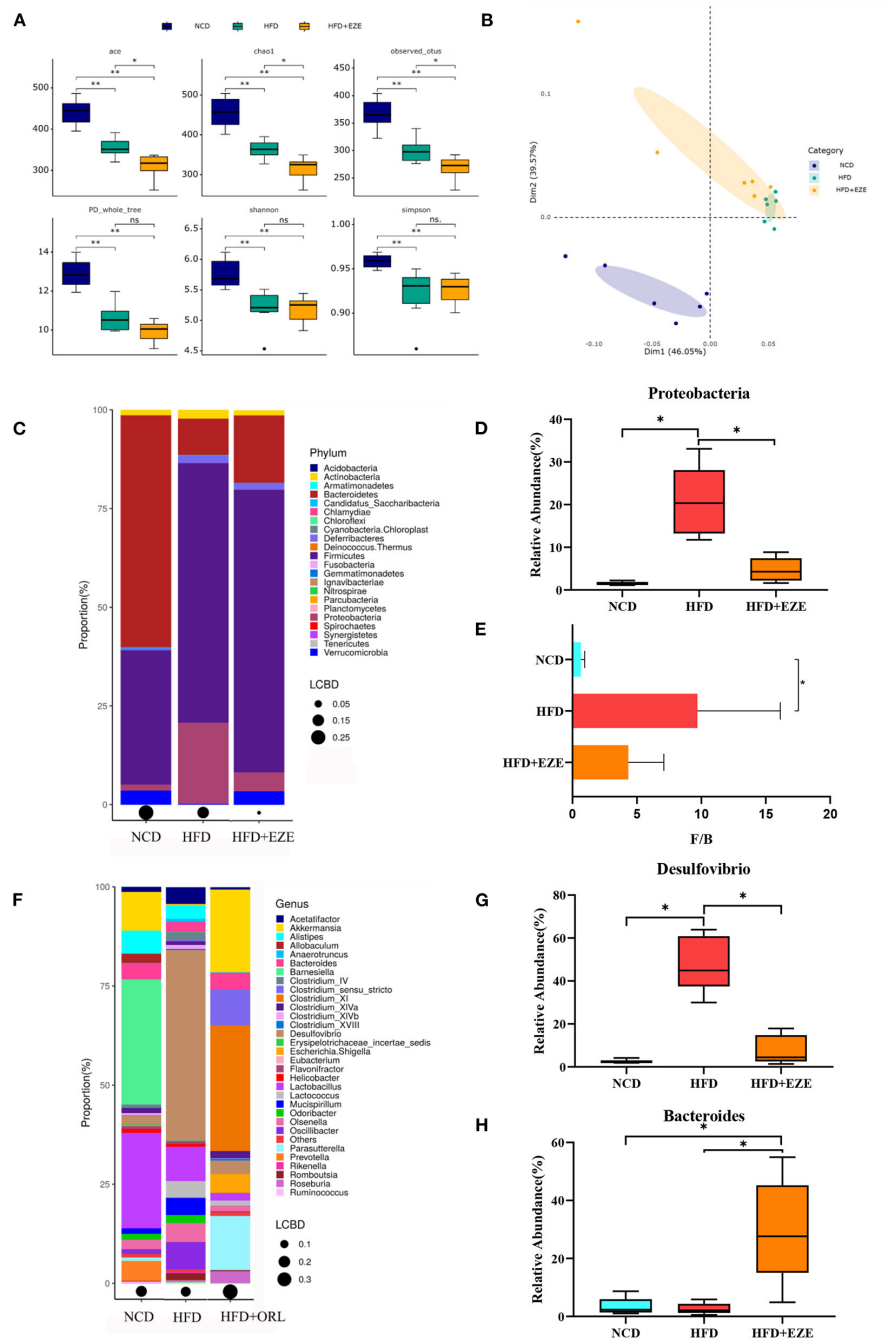
Alterations in gut microbiota before and after ezetimibe intervention. **(A)** The α-diversity of fecal microbiota in mice. **(B)** Beta-diversity was visualized by PCoA analysis. **(C)** Bar plot of gut microbiota composition at the phylum level. **(D)** The relative abundance of *Proteobacteria* was significantly decreased after intervention by Metastas analysis. **(E)** *Firmicutes*-to-*Bacteroidetes* ratio (*F*/*B*) of mice. **(F)** Bar plot of gut microbiota composition at the genus level. **(G,H)** The relative abundance of *Desulfovibrio* was significantly decreased and that of *Bacteroides* was significantly increased after ezetimibe intervention by Metastas analysis. *p < 0.05. LCBD, Local contributions to beta-diversity, LCBD values represent the degree of uniqueness of the sampling units in terms of community composition. NCD, normal diet; HFD, high-fat diet; HFD+ORL, high-fat diet and intervention of orlistat; *n* = 6 per group.

After ezetimibe intervention, the ACE, Chao1, and Observed_OTUs indexes continued to decline (Figure 6A). The PCoA analysis based on the weighted Unifrac distance showed that principal component 1 separated the NCD group from the HFD group, and its interpretation was 46.05%, whereas principal component 2 separated the NCD group from the other two groups, and the axis contribution rate was 39.57%. Among them, the area where the HFD and HFD+EZE groups gather overlapped (Figure 6B). The PERMANOVA analysis showed that the beta-diversity of the HFD group was significantly different from that of the NCD group, while there was no significant difference between the HFD+EZE group and the other two groups (Table 2).

**Table 2:** PERMANOVA analysis of gut microbiota based on weighted Unifrac distance.

**Groups compared**	***P*-values**	***P*-adjust**
HFD+EZE vs. HFD	0.03	0.06
HFD+EZE vs. NCD	0.17	0.17
HFD vs. NCD	0.01	0.02 *

* P < 0.05. NCD, normal diet; HFD, high-fat diet; HFD+ORL, high-fat diet and intervention of orlistat; subject to the adjusted P-value, n = 6 per group.

After 8 weeks of ezetimibe intervention, at the phylum level, the five main bacterial phyla of the HFD+EZE group were *Firmicutes, Bacteroidetes, Proteobacteria, Verrucomicrobia*, and *Deferribacteres* (Figure 6C). Compared to the HFD group, only the relative abundance of *Proteobacteria* was significantly reduced (Figure 6D). After HFD intervention, the *F*/*B* of the HFD group increased significantly. There was no difference in the *F*/*B* value between the HFD+EZE and the HFD and NCD groups (Figure 6E).

At the genus level, the five main bacterial genera in the HFD+EZE group were *Bacteroides, Akkermansia, Desulfovibrio, Acetatifactor*, and *Oscillibacter* (Figure 6F). Compared to the HFD group, the relative abundance of *Desulfovibrio* significantly decreased (Figure 6G), and the relative abundance of *Bacteroides* significantly increased in the HFD+EZE group (Figure 6H).

### Correlation between body weight, FBG, OGTT, serum lipid, and orlistat-responded microbes

**Figure 7:**
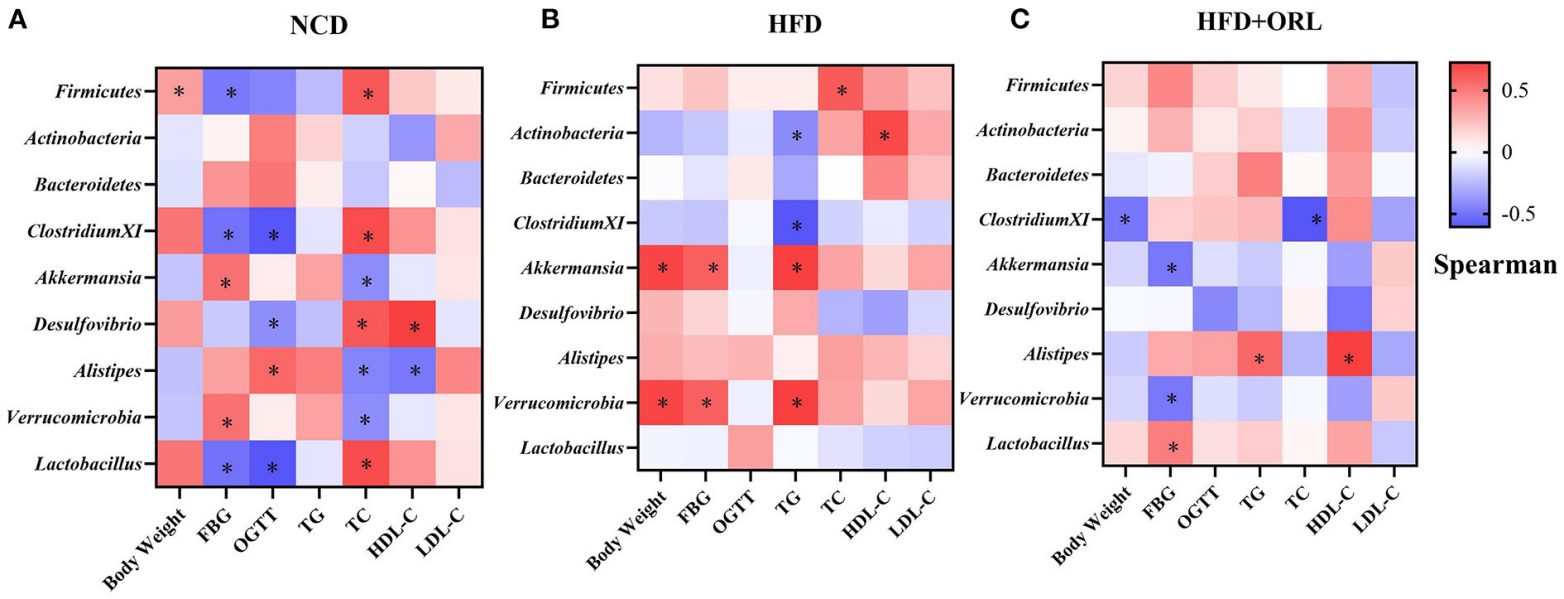
Correlations between orlistat responsive core microbes and phenotypes. **(A)** Heatmap of the correlations between orlistat responsive core microbes and phenotypes in the NCD group. **(B)** Heatmap of the correlations between orlistat responsive core microbes and phenotypes in the HFD group. **(C)** Heatmap of the correlations between orlistat responsive core microbes and phenotypes in the HFD+ORL group. * Indicates that the correlations are statistically significant. Blue indicates the negative correlations, while red indicates the positive correlations. FBG, fasting blood glucose; OGTT, oral glucose tolerance test; TG, triglyceride; TC, total cholesterol; LDL-C, low-density lipoprotein-cholesterol; HDL-C, high-density lipoprotein-cholesterol.

In the NCD group, *Firmicutes* was positively correlated with the body weight and TC and negatively correlated with FBG, *Clostridium XI* was negatively correlated with FBG and OGTT, *Akkermansia* was positively correlated with FBG and negatively correlated with TC, *Desulfovibrio* was positively correlated with TC and HDL-C and negatively correlated with OGTT, *Alistipes* was positively correlated with OGTT and negatively correlated with TC and HDL-C, *Verrucomicrobia* was positively correlated with FBG and negatively correlated with TC, and *Lactobacillus* was positively correlated with TC and negatively correlated with FBG and OGTT (Figure 7A). In the HFD group, *Firmicutes* was positively correlated with TC, *Actinobacteria* was positively correlated with HDL-C and negatively correlated with TG; *Clostridium XI* was negatively correlated with TG; *Akkermansia* was positively correlated with body weight, FBG, and TG; and *Verrucomicrobia* was positively correlated with body weight, FBG, and TG (Figure 7B). In the HFD+ORL group, *Clostridium XI* was negatively correlated with body weight and TC, *Akkermansia* was negatively correlated with FBG, *Alistipes* was positively correlated with TG and HDL-C, *Verrucomicrobia* was negatively correlated with FBG, and *Lactobacillus* was positively correlated with FBG (Figure 7C).

### Correlation between body weight, FBG, OGTT, serum lipid, and ezetimibe-responded microbes

**Figure 8:**
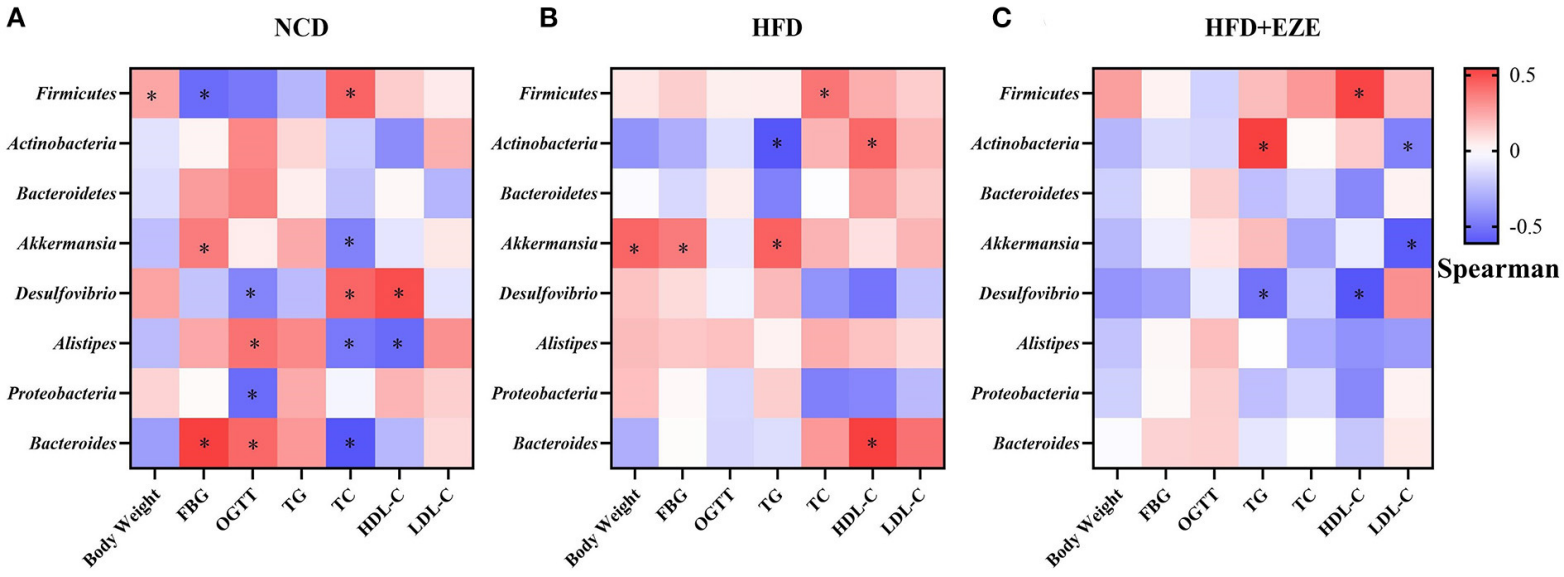
Correlations between ezetimibe responsive core microbes and phenotypes. **(A)** Heatmap of the correlations between ezetimibe responsive core microbes and phenotypes in the NCD group. **(B)** Heatmap of the correlations between ezetimibe responsive core microbes and phenotypes in the HFD group. **(C)** Heatmap of the correlations between ezetimibe responsive core microbes and phenotypes in the HFD+EZE group. *Indicated that the correlations were statistically significant. Blue indicated the negative correlations, while red indicated the positive correlations. FBG, fasting blood glucose; OGTT, oral glucose tolerance test; TG, Triglyceride; TC, total cholesterol; LDL-C, low-density lipoprotein-cholesterol; HDL-C, high-density lipoprotein-cholesterol.

In the NCD group, *Proteobacteria* was negatively correlated with OGTT; *Bacteroides* was positively correlated with FBG and OGTT and negatively correlated with TC (Figure 8A). In the HFD group, *Bacteroides* was positively correlated with HDL-C (Figure 8B). In the HFD+EZE group, *Firmicutes* was positively correlated with HDL-C, *Actinobacteria* was positively correlated with TG and negatively correlated with LDL-C, *Akkermansia* was negatively correlated with LDL-C, and *Desulfovibrio* was negatively correlated with TG and HDL-C (Figure 8C).

## Discussion

In this study, HFD was successfully used to induce a typical obesity phenotype. In addition, the gut microbiota of mice fed with an HFD was disordered. Studies have shown that the gut microbiota, combined with factors related to diet and genetic susceptibility or changes in the gut microbiota related to itself, may be an active driver of obesity (Cox and Blaser, 2013; Shapiro et al., 2017). Many experiments on germ-free mice have found that the obese phenotype can be transferred by gut microbes (Fei and Zhao, 2013; Scheithauer et al., 2020). Similar to most studies (Zhou et al., 2021), in this study, the α-diversity of the gut microbiota of the HFD group decreased, leading to disorders in the gut microbiota. Turnbaugh (Turnbaugh et al., 2006) and other studies have shown that obesity is related to the relative abundance of *Bacteroides* and *Firmicutes* in the gut microbiota. Turnbaugh et al. believed that microbial communities with larger *F*/*B* values are more capable of converting heat from food into fat (Turnbaugh et al., 2009). Other data in humans and rodents revealed that obesity was associated with dysbacteria characterized by an enhanced representation of *Firmicutes* and a reduced representation of *Bacteroidetes* (Rooks et al., 2014; Bian et al., 2022). Similarly, in this study, the *F*/*B* value of the HFD group significantly increased, indicating that the HFD transformed the intestinal microbiota of mice into an obesity phenotype. However, there is controversy about the relationship between the *F*/*B* ratio and obesity. Some studies discovered that *F*/*B* was positively correlated with obesity, but other studies did not observe any modifications in this parameter or even reported a decreased *F*/*B* ratio in obese animals and humans (Magne et al., 2020). Therefore, although the present study found that a high-fat diet resulted in a higher *F*/*B* ratio, suggesting a possible association between an increased *F*/*B* and obesity, the association between *F*/*B* and obesity remains to be further studied due to the limitation of animal study.

Fiber fermentation by the gut microbiota yields SCFAs that are either absorbed by the gut epithelium to participate in a variety of physiologic processes or excreted in feces (de la Cuesta-Zuluaga et al., 2018). Several western epidemiological studies have found that the concentration of total SCFAs and SCFAs of several subtypes in feces are positively correlated with the prevalence of obesity (Schwiertz et al., 2010; Patil et al., 2012; Teixeira et al., 2013; Fernandes et al., 2014; Rahat-Rozenbloom et al., 2014; de la Cuesta-Zuluaga et al., 2018). On the contrary, in animal experiments, it was found that an HFD reduced the concentration of AA in feces (Yamamura et al., 2021). In this study, we found that the concentration of SCFA of various subtypes of the tested mice in the HFD group decreased. In light of the previous studies, the results of this study demonstrated again that the fecal SCFAs could be deeply involved in obesity, and the decreased fecal SCFAs might be one of the important pathological features of diet-induced obesity. The decrease in fecal SCFAs may be related to the decrease in the abundance of SCFAs-producing bacteria in the intestine. Bacterial species belonging to the genera *Bacteroides* and *Prevotella* produce acetate and propionate (Koh et al., 2016). The decrease in AA content may be caused by the decrease in the abundance of *Prevotella* and *Akkermansia muciniphila* in this study.

In this study, orlistat significantly reduced the weight gain of mice induced by HFD, significantly reduced the serum TC and HDL-C levels, and improved dyslipidemia. In addition, orlistat also reduced blood glucose and glucose tolerance and improved metabolic disorders in mice. Moreover, the level of adiponectin in the mice of the high-fat diet group was increased, and orlistat decreased the level of adiponectin. Our previous study also found that a high-fat diet can increase serum adiponectin levels in obese mice (Kang et al., 2021). We inferred that this may result from the significant increase in visceral fat caused by the long-term high-fat diet in this study, thereby secreting more adipokine, such as adiponectin. However, the orlistat intervention reversed the abnormal increase in adiponectin levels, which might be a negative feedback phenomenon caused by the significantly reduced visceral fat. These results were well consistent with the previous studies in which orlistat has been demonstrated to exhibit significant anti-obesity effects clinically. One of the key underlying mechanisms is by inhibiting the absorption of dietary fat. However, whether the anti-obesity effects of orlistat are related to the gut microbiota and its metabolites is unclear.

In the Xenical in the Prevention of Diabetes in Obese Subjects Study, a longitudinal study of patients using orlistat, the mean weight loss from baseline was significantly greater with orlistat (5.8 kg) than with placebo (3.0 kg) after 4 years of treatment (Torgerson et al., 2004). In addition, orlistat also decreased blood lipid levels (Son and Kim, 2020). Singh et al. conducted a meta-analysis of all randomized controlled trials ≥1 year with five anti-obesity drugs and found that orlistat significantly reduced body weight (Singh and Singh, 2020). *Firmicutes, Bacteroides*, and *Actinobacteria* have been documented to be associated with obesity (Ke et al., 2020). HFD consumption is linked with a decrease in *Bacteroidetes*, which exerts immunomodulatory effects on the host, and a higher abundance of *Firmicutes*, which plays a role in energy resorption and obesity (Everard et al., 2014). Other studies have demonstrated a higher abundance of *Actinobacteria* in obese subjects (Turnbaugh et al., 2009). Therefore, orlistat reduced the body weight of obese mice, which may be related to the accumulation of dietary fat in the colon and reduced the abundance of *Firmicutes*. However, no significant effect on *Bacteroides* and *Actinomycetes* was found in this study.

In this study, the microbial diversity, dominant bacteria, and fecal SCFAs were altered after orlistat intervention. These results were consistent with Ke et al. who performed an orlistat intervention study in obese mice (Ke et al., 2020). In this study, some bacterial populations and alpha-diversity were modified by orlistat, presenting as increased *Firmicutes* and *Akkermansia* and a reduction of the α-diversity index, and decreased *Desulfovibrio* and *Alistipes*. The α-diversity index reflects the richness and uniformity of the gut microbiota, and the microbial diversity was normally negatively related to the disease occurrence in adulthood. In our previous population study, we found that overweight and obese individuals in Xinjiang had significantly lower alpha-diversity levels of the gut microbiota (Jin et al., 2021). Our results showed that HFD consumption caused significantly decreased microbial diversity and richness, and orlistat treatment further reduced it. This may be due to the fact that the high triglyceride environment in the gut caused by orlistat use is not conducive to the growth of certain gut microbes, which further reduces the alpha-diversity of gut microbiota. Murphy et al. found a greater abundance of *Desulfovibrio* (species) in people who subsequently had diabetes (Murphy et al., 2017). Besides, in a metagenome-wide association study, sulfate-reducing *Desulfovibrio* species were more prevalent in the gut microbiota of Chinese people with T2DM (Qin et al., 2012). *A. muciniphila* is a promising candidate among the next-generation beneficial microbes that have been identified. Indeed, *A. muciniphila* is inversely associated with obesity, diabetes, cardiometabolic diseases, and low-grade inflammation (Cani and de Vos, 2017). In the present study, *Akkermansia* was negatively correlated with FBG after the intervention of orlistat, which indicated *Akkermansia* may be related to the hypoglycemic effect of orlistat. *Alistipes* is a relatively new genus that belongs to the phylum *Bacteroidetes*, which is highly relevant in dysbiosis and disease (Parker et al., 2020). This study found that *Alistipes* decreased after orlistat intervention, the abundance of which was negatively correlated with obesity, lipid, and glucose homeostasis parameters (Garcia-Ribera et al., 2020). Another population study also found that the abundance of *Alistipes* was negatively correlated with body mass index (Lv et al., 2019). In the present study, we found that *Alistipes* was negatively correlated with TC and HDL-C levels in the NCD group and positively correlated with HDL-C levels after the intervention of orlistat. Studies have shown that *Alistipes* is related to certain diseases, such as anxiety, myalgic encephalomyelitis/chronic fatigue syndrome, depression, pervasive developmental disorder not otherwise specified, and colorectal cancer. In contrast, in terms of pathogenicity, it was believed that their presence is correlated with the promotion of healthy phenotypes, such as colitis, autism spectrum disorder, and various liver and cardiovascular fibrotic disorders (Parker et al., 2020).

The BA bacteria are distributed in the colon and cecum, mainly *Firmicutes*, including *Fusobacterium, Eubacterium, Clostridium*, and *Roseburia* (Louis and Flint, 2009). Although the relative abundance of *Firmicutes* decreased after orlistat intervention, *Firmicutes* are still the main bacteria in this study. Besides, this study found that *Clostridium sensu stricto* and *Clostridium XI* significantly increased after orlistat intervention. Moreover, *Clostridium XI* has been found to be negatively correlated with body weight and TC after orlistat intervention. This result indicated that *Clostridium XI* might be a keystone that is associated with the hypolipidemic efficacy of orlistat, which implies that the depletion of this genera might facilitate orlistat to improve lipid metabolism and obesity. A large number of phylogenetic studies based on the 16S rRNA gene sequencing indicated that *Clostridium* should be restricted to *Clostridium I* as the narrow sense *Clostridium* (*Clostridium sensu stricto*) (Galperin et al., 2016). *Clostridium sensu stricto* is sufficiently close to the type species *Clostridium butyricum* (Lawson and Rainey, 2016). Pan et al. found that supplementation with *C. butyricum* can restore the gut microbiota composition and enhance butyrate production in feces (Pan et al., 2021). *Clostridium XI* is the phylogenetic cluster containing *Clostridium difficile* and is considered a harmful bacterium in the intestine. In this study, *Clostridium XI* and *Clostridium sensu stricto* may be the most important factors in increasing BA. Moreover, *Desulfovibrio* has been confirmed to have acetate and butyrate as metabolites (Dubinski et al., 2021). The changes in BA-producing bacteria in this study were complex, manifested as an increase in *Clostridium XI* and *Clostridium sensu stricto* and a decrease in *Desulfovibrio, Clostridium XIVa*, and *Clostridium XVIII*, which ultimately led to an increase in BA in feces. Studies have found that AA and lactic acid can be used by BA-producing bacteria to produce BA (Louis et al., 2004). At the same time, the increase in *Akkermansia* and *Escherichia*/*Shigella* did not cause an increase in acetate in feces, which may also be one of the reasons for the increase in BA. In this study, the increase in AA and CA may result from the combined effects of the gut microbiota, mainly the increase of *Clostridium XI* and *Clostridium sensu stricto*, which still needs further research.

Ezetimibe is a selective intestinal cholesterol absorption inhibitor that acts on the brush border of the small intestinal mucosa, specifically binds to the Niemann-Pick C1-like 1 transporter on the intestinal mucosa, and selectively inhibits the absorption of exogenous cholesterol (Law et al., 2003). In a pilot study, Akira Kurozumi et al. investigated the effects of postprandial lipid abnormalities induced by HFD loading on vascular endothelial function in T2DM and evaluated the effects of ezetimibe on endothelial function. This study found that ezetimibe can potentially inhibit the aggravation of vascular endothelial dysfunction by improving dyslipidemia after HFD loading (Kurozumi et al., 2016). Ezetimibe restored the postprandial dysregulation of lipids but did not affect glucose metabolism in a double-blind randomized crossover trial (Kikuchi et al., 2012). Consistently, in this study, TC and HDL-C levels were decreased by ezetimibe, but TG levels showed no significant difference in obese mice, which were different from the results of other studies (Pandor et al., 2009). However, after ezetimibe intervention, this study did not find a significant difference in body weight, visceral fat, insulin, adiponectin, leptin, blood glucose, and OGTT. Robert Krysiak et al. showed that 14-day ezetimibe treatment induces relatively small changes in fat tissue hormonal function in patients with isolated hypercholesterolemia (Krysiak et al., 2014). In addition, one study showed that 14-day treatment with ezetimibe did not alter circulating adiponectin, resistin, or leptin levels in healthy men (Gouni-Berthold et al., 2008).

One study showed that the gut microbiota could shift the host's cholesterol absorption/synthesis balance (Le Roy et al., 2019). In this study, the α-diversity and predominant bacteria were altered by ezetimibe, showing that the relative abundance of some low-abundance bacteria changed. Similar results were obtained with orlistat intervention, that is, *Desulfovibrio* decreased after ezetimibe intervention, and *Desulfovibrio* was negatively correlated with TG and HDL-C levels after ezetimibe intervention. Almada Caroline et al. found that inactivated probiotics caused a decrease in the abundance of *Bacteroides* (Almada et al., 2021). This study found a higher abundance of *Bacteroides*, which was negatively correlated with the expression levels of serum miR-122-5p that was found to be significantly enriched in glucose metabolism-related signaling pathways (Li et al., 2020). An experiment on obese rats induced by HFD and treated with diabetes drugs found that berberine can increase the *Bacteroides* content, a class of putative SCFA-producing bacteria (Zhang et al., 2015). However, there was no significant difference in SCFAs, in accordance with the findings of human research (Jin et al., 2021). Besides, this study did not find significant effects of ezetimibe on body weight and accumulation of visceral fat, blood glucose, insulin, leptin, and adiponectin. This is probably because ezetimibe only caused a few low-abundance bacterial changes and had little apparent effects on mouse glucose and lipid metabolism, but only affected the cholesterol metabolism pathway. A recent study found that an HFD promoted the flourishment of *Proteobacteria* (Chang et al., 2017). In addition, a group of patients with diabetic cardiovascular complications and a group of T2DM patients had a significant increase in the proportion of *Proteobacteria* (Larsen et al., 2010). In this study, ezetimibe decreased *Proteobacteria*, which was not significantly different from the NCD group. Another study found that intestinal epithelial cells (IECs) responded to infection by activating Srebp2 and the cholesterol biosynthetic pathway, resulting in higher fecal cholesterol levels and a bloom of *Proteobacteria* (Berger et al., 2017). This may be related to the high-cholesterol environment in the intestine. The negative feedback regulation of a large amount of cholesterol accumulation in the intestine inhibited IEC activation of cholesterol synthesis, inhibited *Proteobacteria* proliferation, and reduced the serum cholesterol content.

In conclusion, the 20-week HFD intervention caused a typical obesity phenotype, damaged gut microbiota, and decreased SCFA production. Orlistat and ezetimibe intervention could characteristically alleviate the weight gain, serum TC, HDL-C, and dyslipidemia induced by HFD. These different anti-obesity effects might, at least partly, result from their different abilities to reduce the phylum *Firmicutes* and increase SCFA-producing bacteria, eventually leading to an increase in the gut BA content. The enhanced anti-obesity effects of orlistat might result from its stronger ability to alter gut microbiota and SCFAs, at least partly. However, some specific drug-related microorganisms potentially related to glucose and lipid metabolism need to be further studied.

## Limitation

The limitation of this work lies in the data analysis approach. The data analysis uses QIIME instead of QIIME2. No one is maintaining QIIME to ensure the validity of results.

## Data availability statement

The data presented in the study are deposited in the Sequenced Read Archive (SRA) repository, accession number (PRJNA816265).

## Ethics statement

The animal study was reviewed and approved by Experimental Animal Center, West China Second Hospital, Sichuan University.

## Author contributions

HT, FH, JJ, RC, and JW designed the study. JW and JJ performed the experiments, analyzed the data, and drafted the manuscript. RC, XS, ZM, YL, QZ, YR, and YX participated in partial experiments and data collection process. FH, RC, and JW revised the manuscript. All authors reviewed the manuscript. All authors agreed to have two corresponding authors.

## Funding

This work was supported by the Basic and clinical research of endocrine and metabolic diseases, big data, translational medicine, and precision medicine (Grant Number: ZYGD18022).

## Conflict of interest

The authors declare that the research was conducted in the absence of any commercial or financial relationships that could be construed as a potential conflict of interest.

## Publisher's note

All claims expressed in this article are solely those of the authors and do not necessarily represent those of their affiliated organizations, or those of the publisher, the editors and the reviewers. Any product that may be evaluated in this article, or claim that may be made by its manufacturer, is not guaranteed or endorsed by the publisher.
